# Advancing Medical Applications of Cancer Nanotechnology: Highlighting Two Decades of the NCI'S Nanotechnology Characterization Laboratory Service to the Research Community

**DOI:** 10.1002/wnan.70020

**Published:** 2025-06-03

**Authors:** Rachael M. Crist, Yechezkel Barenholz, Ahuva Cern, Kate N. Clark, Pieter R. Cullis, Cheryl Dean, Neil Desai, Mauro Ferrari, Matthieu Germain, Carmen A. Giacomantonio, Emma Grabarnik, Piotr Grodzinski, Atara Hod, Barry E. Kennedy, Ruvanthi N. Kularatne, Glen S. Kwon, Emmanuel Loeb, Erin B. Noftall, Len Pagliaro, Morteza Rasoulianboroujeni, Alexander Roth, Darren Rowles, Kulbir Singh, Nicole F. Steinmetz, Zhanna Yehtina, Yao Zhang, Daniel Zilbersheid, Jeffrey D. Clogston, Stephan T. Stern, Marina A. Dobrovolskaia

**Affiliations:** ^1^ Nanotechnology Characterization Laboratory, Cancer Research Technology Program, Frederick National Laboratory for Cancer Research Sponsored by the National Cancer Institute Frederick Maryland USA; ^2^ Laboratory of Membrane and Liposome Research, Department of Biochemistry The Hebrew University of Jerusalem Jerusalem Israel; ^3^ Department of Pathology, Faculty of Medicine Dalhousie University Halifax Nova Scotia Canada; ^4^ Department of Biochemistry and Molecular Biology University of British Columbia Vancouver British Columbia Canada; ^5^ Aanastra Los Angeles California USA; ^6^ Department of Pharmaceutics School of Pharmacy, University of Washington Seattle Washington USA; ^7^ Nanobiotix Paris France; ^8^ Department of R&D Sona Nanotech, Inc. Halifax Nova Scotia Canada; ^9^ Department of Surgery, Faculty of Medicine Dalhousie University Halifax Nova Scotia Canada; ^10^ Nanodelivery Systems and Devices Branch, Cancer Imaging Program, Division of Cancer Treatment and Diagnosis National Cancer Institute, National Institutes of Health Rockville Maryland USA; ^11^ Pharmaceutical Sciences Division School of Pharmacy, University of Wisconsin‐Madison Madison Wisconsin USA; ^12^ Patho‐Logica Ness Ziona Israel; ^13^ Aiiso Yufeng Li Family Department of Chemical and Nano Engineering University of California San Diego California USA; ^14^ Department of Bioengineering University of California San Diego California USA; ^15^ Department of Radiology University of California San Diego California USA; ^16^ Shu and K.C. Chien and Peter Farrell Collaboratory, University of California San Diego California USA; ^17^ Center for Nano‐ImmunoEngineering University of California San Diego California USA; ^18^ Center for Engineering in Cancer Institute of Engineering Medicine, University of California San Diego California USA; ^19^ Moores Cancer Center University of California San Diego California USA; ^20^ Institute for Materials Discovery and Design, University of California San Diego California USA; ^21^ School of Biomedical Engineering, University of British Columbia Vancouver British Columbia Canada; ^22^ Michael Smith Laboratories University of British Columbia Vancouver British Columbia Canada

**Keywords:** cancer, nanomedicine, nanotechnology

## Abstract

The Nanotechnology Characterization Laboratory (NCL) is a US federally funded resource providing characterization and expertise to the cancer nanomedicine research community. Founded as a formal partnership among the US National Cancer Institute (NCI), the US Food and Drug Administration (FDA), and the US National Institute of Standards and Technology (NIST), the NCL has spent two decades developing a one‐of‐a‐kind service with broad multidisciplinary expertise to meet the needs of a rapidly evolving drug development field. To mark the 20th anniversary of the lab's founding, the NCL hosted a symposium to highlight the achievements of the cancer nanomedicine field, showcase novel, next‐generation nanotechnology research, and discuss future priorities to enable continued growth in combating cancer and the complexities associated with treating a disease that continues to take millions of lives annually. The discussion topics from this event are summarized.

## Graphical abstract

The field of cancer nanomedicine has seen tremendous advancements over the last two decades, not only in the nanotechnologies themselves, but in the applications and uses of these novel nanomaterials and the characterization techniques employed to better understand both their physicochemical and biological properties. AI, artificial intelligence; QSAR, quantitative structure–activity relationship.

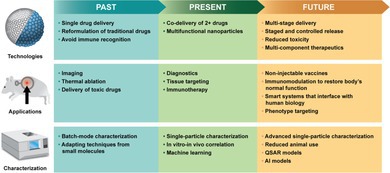

## 1 Introduction

The Nanotechnology Characterization Laboratory (NCL) was founded in 2004 as a formal partnership among the US National Cancer Institute (NCI), the US Food and Drug Administration (FDA), and the US National Institute of Standards and Technology (NIST) with the mission of advancing the science of cancer nanotechnology. During this period, the NCL developed nearly 100 assays to thoroughly characterize a nanoparticle's physicochemical, immunological, toxicological, and pharmacokinetic properties, established collaborations with several hundred academic, government, and industrial organizations from around the world, and characterized more than 1000 nanoparticles, including every nanotechnology platform being used in biomedical research. This work—and these partnerships—have revealed many unique physicochemical and biological correlations and aided in the advancement of nearly two dozen novel cancer nanomedicine products into or through human clinical trials.

In 2024, the NCL observed its 20th anniversary, marked with a symposium featuring presentations from some of the field's most well‐respected scientists. Topics included historical perspectives on the nanomedicine drug development field, national priorities for nanotechnology research, current research initiatives in cancer nanomedicine, and highlights and trends from the NCL's two decades of cancer nanomedicine characterization. The presentations and discussions from the event are summarized herein to further disseminate the topics and dialogue from the symposium to the global nanotechnology research community.

## 2 Nanomedicine Achievements and Priorities

Early nanomedicine research, to a large extent, focused on improving the delivery of existing, approved drugs and decreasing any associated toxicities. For example, the formulation of the cytotoxic agent doxorubicin using a PEGylated liposome (Doxil) reduced the cardiotoxicity associated with the administration of the free drug. Similarly, the formulation of paclitaxel with nanoparticle albumin (nab) (Abraxane) reduced hypersensitivity reactions, that is, complement activation related pseudoallergy (CARPA), experienced by patients receiving Cremophor‐EL formulated paclitaxel (Taxol). Today, with new, novel platform technologies being developed and a deeper understanding of cancer biology and its relationship with the immune system, nanomedicine research has swelled beyond the formulation of traditional cytotoxic agents.

### 2.1 Achievements in the Drug Development Field—A Clinical Journey

**FIGURE 1:**
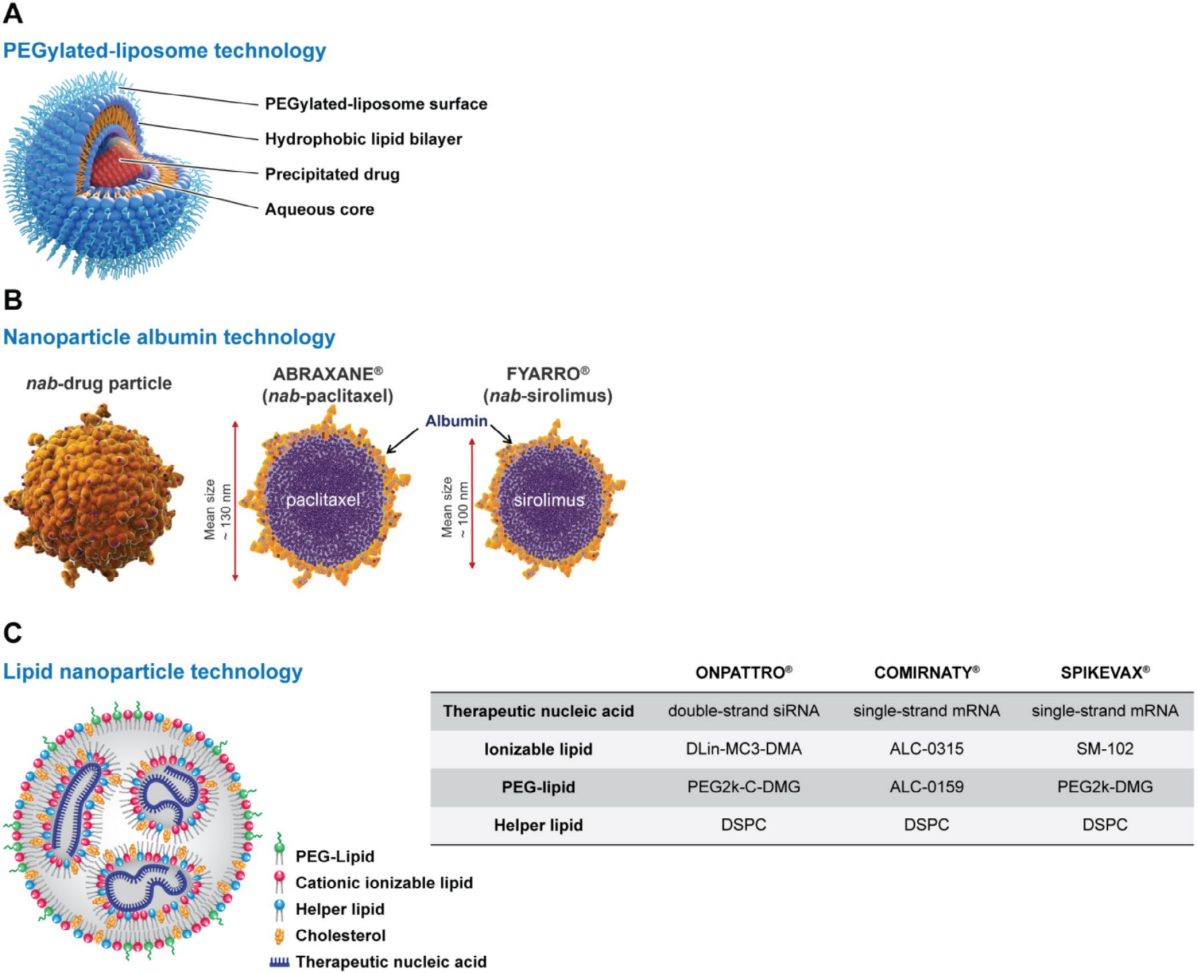
Schematic illustrations of the (A) PEGylated liposomal technology used in the development of Doxil (figure adapted from Barenholz 2012), (B) nab‐technology used in the development of Abraxane and Fyarro, and (C) lipid nanoparticle technology used in the development of Onpattro, Comirnaty, and Spikevax. The LNP schematic was reproduced from (Kularatne et al. 2022) (https://creativecommons.org/licenses/by/4.0/); the compositional information in the table was adapted from (Suzuki and Ishihara 2021).

Liposomes are considered to be one of the earliest nanotechnology platforms used for drug delivery, and considering the number of clinically approved liposomal drugs, many would consider liposomes one of the most successful platforms. Doxil (PEGylated liposomal doxorubicin), developed in the laboratories of Yechezkel Barenholz and Alberto Gabizon, is considered to be the first FDA‐approved nanomedicine; initial approval in 1995 was for treatment of AIDS‐related Kaposi's sarcoma, followed later by approval for treatment of breast and ovarian cancer and multiple myeloma (Barenholz 2012). Barenholz describes the development process for Doxil in an earlier review article (Barenholz 2012), notably stating, “Each component matters and contributes to the optimized performance” (Figure 1A). From the incorporation of a PEGylated lipid component in the lipid membrane which helps avoid detection by the immune system, thereby enabling prolonged circulation times, to the use of an ammonium sulfate gradient which affords a stable precipitation of the drug in the liposome interior, to the tuned size of the particle which allows extravasation of the tumor vasculature, the precisely designed formulation has served as a model for later liposomal developments.

With the success of Doxil, liposomal technology has been a mainstay of nanotechnology‐centric drug delivery. There have been at least five generic versions of Doxil approved by the FDA since 2013. In oncology, Onivyde (PEGylated liposomal irinotecan) and Vyxeos (liposomal cytarabine and daunorubicin) are two additional commercially approved liposomal treatments. Onivyde was approved in 2015 for the treatment of pancreatic cancer, and Vyxeos was approved in 2017 for the treatment of acute myeloid leukemia. In total, there are more than a dozen liposomal drugs approved by the FDA across a variety of therapeutic indications (Bulbake et al. 2017; Gu et al. 2023), validating the platform as a key vehicle in the continued efforts to improve drug delivery.

Another early nanomedicine success was Abraxane, which utilized nanoparticle albumin‐bound (nab) technology to formulate paclitaxel (Figure 1B). The legacy formulation of paclitaxel (Taxol), marketed in 1992 by Bristol Myers Squibb, used Cremophor EL for intravenous administration, caused severe hypersensitivity reactions, including anaphylaxis and a high (38%) mortality rate (Irizarry et al. 2009) in addition to severe myelosuppression and neuropathy. The NCL subsequently confirmed in laboratory tests that Taxol—but not Abraxane—caused severe complement activation, explaining the clinical hypersensitivity observations (Dobrovolskaia and McNeil 2013a). Nab‐paclitaxel improved tumor penetration through a caveolar albumin transport mechanism (Predescu et al. 2004) while greatly reducing adverse effects. It became the first protein‐based nanoparticle drug approved for metastatic breast cancer (2005), non‐small cell lung cancer (2012), and pancreatic cancer (2013), with additional immune‐oncology therapy approvals (2018–2020) (ABRAXANE package insert 2020).

Following the success of Abraxane, Desai, who pioneered its development, leveraged the albumin‐bound technology to develop Fyarro (nab‐sirolimus). Fyarro addressed the limitations of mTOR inhibitors (sirolimus, everolimus) including poor bioavailability and low mTOR target inhibition. Nab‐sirolimus demonstrated significantly higher tumor accumulation, superior efficacy, and lower toxicity in preclinical studies (Hou et al. 2019). Fyarro's clinical development focused on advanced malignant perivascular epithelioid cell tumors (PEComa), an mTOR driven rare sarcoma (~100–300 U.S. incidence) (Bleeker et al. 2012) with no approved treatments. In the AMPECT trial for advanced PEComa, nab‐sirolimus achieved a 39% overall response rate and mean duration of response of 39.7 months, with a high disease control rate and manageable toxicities (Wagner et al. 2021). Fyarro's characterization portfolio—including data generated in the NCL Assay Cascade—was submitted in May 2021, and the formulation was approved by the FDA in November 2021 as the first and only treatment for advanced malignant PEComa (FYARRO package insert 2021).

More recently, immense progress has been made in the field of lipid nanoparticles (LNPs) for nucleic acid delivery (Figure 1C), forged by research findings from Pieter Cullis and coworkers. The 2018 FDA approval of Onpattro (Adams et al. 2018; Wood 2018), an LNP‐based siRNA formulation for the treatment of polyneuropathies caused by the hereditary disease transthyretin‐mediated amyloidosis (hATTR), provided clinical validation of the LNP system for in vivo delivery of nucleic acid macromolecules (Akinc et al. 2019). This achievement was further reinforced by the worldwide impact of the LNP mRNA COVID‐19 vaccines (Baden et al. 2021; Polack et al. 2020) that played a significant role in alleviating the global pandemic. LNP‐based delivery systems have many advantages compared to viral and other non‐viral vectors, including improved safety profiles, ability to re‐dose, versatility of desired cargo, ease of scale‐up, and cost‐effective manufacturing processes (Cullis and Hope 2017; Witzigmann et al. 2020). Additionally, LNP mRNA systems have high transfection competency across several administration routes, including intravenous, intramuscular, intradermal, subcutaneous, intraperitoneal, and intratracheal injection (Pardi et al. 2015).

LNP delivery technology has now been applied to the development of many other nucleic acid‐based drugs, leading to over 60 vaccines and therapeutics that are currently in clinical development or have already obtained regulatory approval (Cullis and Felgner 2024). Applications of LNP RNA systems to achieve gene editing, as well as methods to achieve tissue‐ and cell‐specific transfection, appear imminent (Kularatne et al. 2022). The LNP technology is rapidly enabling the full potential of gene therapies to treat most human diseases, including infectious diseases (Kackos et al. 2023; Kawai et al. 2025; Mu et al. 2022; Saunders et al. 2021), cancer (Meulewaeter et al. 2024; Qiu et al. 2023; Ramos da Silva et al. 2023), rare diseases (Koeberl et al. 2024; Strilchuk et al. 2024; Yu et al. 2022), as well as more common diseases such as cardiovascular disease (Musunuru et al. 2021; Soroudi et al. 2024). Notable achievements include transfection of T cells (Billingsley et al. 2024; Rurik et al. 2022) to enable in vivo CAR‐T cell therapies or transfection of hematopoietic stem cells (HSCs) in bone marrow (Breda et al. 2023) for the treatment of disorders ranging from leukemia or lymphoma to sickle cell anemia.

### 2.2 US National Priorities

Nanotechnology—not just nanomedicine—has been a national priority for the US since the signing of the 21st Century Nanotechnology Research and Development Act by former President George W. Bush in 2003. With this, the National Nanotechnology Initiative (NNI) and National Nanotechnology Coordination Office (NNCO) were formed, alongside other working groups and committees across various health, safety, and environmental focuses. The NNI sets national priorities for nanotechnology in multiple disciplines, including health, artificial intelligence, national security, and climate preservation, among others (National Nanotechnology Coordination Office 2024). The NNI's focus on using nanotechnology to promote health has remained at the forefront of their efforts for years, with more than 40% of the fiscal year 2025 proposed $2.2 billion budget going to the National Institutes of Health (NIH), following similar trends from years past (National Nanotechnology Coordination Office 2024). The Office of Science and Technology Policy and Office of Management and Budget outlined a goal to “achieve better health outcomes for every person” as part of the 2025 budget priorities (Office of Management and Budget and Office of Science and Technology Policy 2023). Directly related to cancer nanomedicine, the report calls for support of former President Joseph Biden's Cancer Moonshot program. Reducing both suffering and death from cancer has been a top priority since former President Richard Nixon signed The National Cancer Act into law in 1971. There has, undoubtedly, been great achievements in this regard in the last 50 years, but the sustained prevalence of cancer reaffirms the need for renewed support. The memo calls for improved early detection strategies, efforts to promote prevention, and development of novel therapies—an area specifically where cancer nanomedicine research and development is and can continue to contribute. Other health‐specific activities called out included mitigating microbial resistance, enhancing preparedness for infectious disease outbreaks, supporting at‐risk communities, improving health equity, advancing efforts for rare diseases, and reducing environmental impacts (Office of Management and Budget and Office of Science and Technology Policy 2023).

**20th Anniversary of the 21st Century Nanotechnology Research and Development Act**.

The National Nanotechnology Coordination Office (NNCO) also recently celebrated the 20th Anniversary of the 21st Century Nanotechnology Research and Development Act, signed into law by former President George W. Bush on December 3, 2003. The symposium, “Enabling the Nanotechnology Revolution,” featured discussions not only on medicine, but also engineering, environmental safety, manufacturing, education, and more. A full video archive of the NNCO's March 2024 event can be found here: https://www.nano.gov/anniversarysymposium.

### 2.3 US National Cancer Institute Efforts

The NCI established the Alliance for Nanotechnology in Cancer (ANC) in 2005 to capitalize on emerging innovation in the areas of nanomaterials and nanodevices and their potential utility in cancer research and care. The program's goal was to support discovery and applied research with the added long‐term goal of producing clinically useful outcomes. Cancer nanotechnology is a multi‐disciplinary field; accordingly, the Alliance targeted a multi‐disciplinary community of biologists, clinicians, chemists, and engineers to leverage innovation and experience originating from different research backgrounds.

The Alliance, in its original incarnation, focused on the development of technology platforms that were seeking appropriate cancer applications. Since these initial years, the program has matured and evolved from technology‐focused to oncology application‐focused and defined relevant biological and clinical problems, which served as a driver for implementing suitable nanotechnologies. Subsequently, several technologies developed under ANC funding have reached a level warranting the initiation of clinical trials (Hartshorn et al. 2018; Hartshorn et al. 2019).

The ANC network involved multiple synergistic NCI‐funded initiatives for large research centers, smaller research projects, multidisciplinary training awards, as well as support of the NCL. The Centers of Cancer Nanotechnology Excellence (CCNEs), which operated for 15 years (2005–2020), were focused on integrating nanotechnology and cancer research to develop solutions that are clinically relevant (Grodzinski 2019). They provided infrastructure and translational support to the ANC network. Currently, the program funds R01 grants via two funding opportunities: the Innovative Research in Cancer Nanotechnology (IRCN) and Toward Translation of Nanotechnology Cancer Interventions (TTNCI) (Innovative Research in Cancer Nanotechnology 2025; Toward Translation of Nanotechnology Cancer Interventions 2025). These two announcements cover opposite ends of the funding spectrum for cancer nanotechnology; the former is focused on mechanistic studies contributing to the fundamental understanding of nanoparticle and nano‐devices design rules and mechanisms governing their in vivo interactions, while the latter paves the way for late preclinical evaluations, improving entry of nanotechnology cancer interventions into GMP/GLP evaluations and long‐term into human studies. Many of these ANC‐funded awards have benefited from evaluation of their nanomaterials at the NCL, aiding the selection of promising nano‐therapeutic and diagnostic candidates for further development.

### 2.4 Novel Developments

Combating the unique intricacies of cancer requires equally unique approaches. The NCL has worked with hundreds of researchers around the globe, spanning the plethora of nanotechnology platforms used in cancer research for applications such as drug delivery, imaging, immunotherapy, radiotherapy, and more. Highlighted here are five select projects using novel nanotechnology‐based approaches to tackle cancer: a plant virus that aims to reprogram the immune response toward tumors; a non‐drug‐loaded liposome to occupy liver and spleen macrophages, thereby enabling greater drug accumulation in target sites; gold nanorods for enhancing radiation therapy; a polymeric prodrug formulation aimed at improving the therapeutic index of one of the most widely utilized chemotherapeutic agents, paclitaxel; and a liposomal formulation of an angiotensin receptor blocker designed to normalize the tumor microenvironment (TME) and improve the activity of immune checkpoint inhibitors.

#### 2.4.1 Cowpea Mosaic Virus as an Immunotherapy Candidate

In 2015, Steinmetz (UC San Diego) and Fiering (Dartmouth College) discovered that a plant virus—cowpea mosaic virus (CPMV)—stimulates potent anti‐tumor immunity when applied intratumorally (Lizotte et al. 2016). CPMV is a 30 nm‐sized nanoparticle forming an icosahedral capsid packaging a positive‐sense bipartite ssRNA genome (Bancroft 1962; Bruening and Agrawal 1967; Wu and Bruening 1971). The plant virus nanoparticles are produced through plant molecular farming using black‐eyed peas. Intratumoral CPMV stimulates potent, systemic, and durable anti‐tumor immunity in murine tumor models (Koellhoffer and Steinmetz 2022; Lizotte et al. 2016; Mao et al. 2021; Mao et al. 2022; Shukla et al. 2020; Wang et al. 2019; Wang and Steinmetz 2020) and in canine cancer patients (companion pets) with spontaneous tumors, significantly improving tumor‐free survival (Alonso‐Miguel et al. 2022; Hoopes et al. 2018; Valdivia et al. 2023). CPMV overcomes immunosuppression within the TME, launching both local and systemic adaptive anti‐tumor immunity, thereby suppressing both local and distant metastases (abscopal effect).

Enabled through research grants supported through the ANC initiative, a CPMV lead candidate for translational consideration was developed. In collaboration with the NCL, CPMV's mechanism of action was validated, and its pharmacology was documented. This study highlighted CPMV's potential not only as an intratumoral agent but also suggested CPMV may induce anti‐tumor immunity after systemic administration. Indeed, it was recently shown that systemic CPMV administration before tumor challenge protects mice from the onset of tumor growth (Chung et al. 2024). Longitudinal analysis using a metastatic mouse model of colon cancer with intraperitoneal metastases demonstrated that the “immunoprevention” effect was maintained over a 14‐day window. The CPMV “immunoprevention strategy” was also demonstrated in mouse models of i.p. disseminated ovarian cancer and lung metastases from intravenous challenge with melanoma cells or breast cancer cells. In a head‐to‐head comparison of the efficacy of CPMV against other immunomodulatory adjuvants, CPMV demonstrated superior protection against tumor challenge compared to STING and TLR7 agonists (Chung et al. 2024). Collectively, these studies indicate that CPMV acts as a training agent and induces heterologous protection against tumor challenge. From a practical perspective, CPMV holds great potential as an intratumoral agent when used as a neoadjuvant or systemic adjuvant therapy post‐surgery to prevent recurrence and outgrowth of metastatic disease.

#### 2.4.2 Nanoprimer Technology to Increase Systemic Bioavailability

Despite progress in the design of therapies administered intravenously, the liver remains one of the main challenges for treatment delivery. Hepatic clearance is responsible for the low delivery of treatment to the target site and the limited efficacy outcome. Moreover, the unintended liver distribution could cause harmful side effects. To address this challenge, Nanobiotix has developed the Curadigm platform—Nanoprimer, a technology aiming to shift the balance of therapeutic agents' bioavailability and toxicity. The platform is designed to decrease therapeutic agents' liver trapping, affording increased systemic bioavailability for optimal accumulation in target tissues (Germain et al. 2018). Nanoprimer is an engineered, biocompatible liposome that transiently and specifically occupies the liver clearance pathways responsible for sub‐optimal therapeutic bioavailability and is intended to be administered just before the treatment. By interacting specifically with the receptors of the mononuclear phagocytic system cells, the Nanoprimer enables a temporary reduction of drug clearance.

Preliminary results have shown a good safety profile for the Nanoprimer. In vitro studies have shown the Nanoprimer does not activate the complement system and does not lead to cytokine‐mediated immune reactions. Further, multiple proof‐of‐concept studies have shown the ability of the Nanoprimer to improve the efficacy of various innovative therapeutics, including nanomedicines such as RNA‐loaded lipid nanoparticles (siRNA‐LNP). Evaluation in a mouse model showed addition of the Nanoprimer during the treatment leads to a 40% reduction in siRNA‐LNP accumulation in the liver, correlating to an 8‐fold increase in the systemic bioavailability of the siRNA‐LNP 1 h after i.v. injection (Saunders et al. 2020). In a follow‐on study evaluating the impact of the Nanoprimer on the efficacy of siRNA‐LNP, Nanoprimer addition was shown to double the tumor growth inhibition generated by the siRNA‐LNP therapeutic alone. Another proof of concept looked at the effect of Nanoprimer addition to oncolytic viruses, which see their use by i.v. administration highly limited by the liver. Using herpes simplex virus 1 and a murine B‐16 tumor model, addition of the Nanoprimer led to a 10‐fold increase in viral copy number in the tumor, opening possibilities for cancer treatment by i.v.‐administered oncolytic viruses. Finally, in a study conducted with the NCL, the Nanoprimer was shown to improve the accumulation of a scavenger receptor A1‐targeted poly(L‐lysine succinylated) (PLS) based therapeutic in tumor‐associated macrophages (TAMs) by threefold. The polymer was developed to deliver various cargos to macrophages and other myeloid cells (Stevens et al. 2020). Since the Nanoprimer accumulates preferentially in liver and spleen macrophages, liver and spleen uptake of the polymer is decreased, allowing for greater polymer accumulation in TAMs, improving PLS‐based immunotherapies.

Altogether, these preclinical findings are very encouraging for the continued development of the Nanoprimer technology. The ubiquitous mode of action of the Nanoprimer allows its application to a broad spectrum of drugs ranging from nanomedicine to biologics and has the potential to redefine the benefit/risk ratio of drugs, improving their clinical outcomes and treatment value.

#### 2.4.3 Gold Nanorods Induce Immunogenic Cell Death via Intra‐Tumoral Hyperthermia

Sub‐ablative hyperthermia (tissue temperature of ~45°C) is an increasingly established adjuvant or neo‐adjuvant option in cancer treatments, with mechanisms including immune modulation, selective cancer cell death, and vascular changes leading to enhanced tumor perfusion. Traditional hyperthermia applications are largely focused on ablation (> 55°C), resource‐intensive, and often associated with patient morbidity, limiting their clinical accessibility. Gold nanorods (GNRs) offer a precise, minimally invasive tool for targeting sub‐ablative hyperthermia to the tissues of interest using near‐infrared (NIR) light to excite vibrational energy in the GNRs and deliver targeted hyperthermia therapy (THT) with precision. THT induces controlled tumor heating, promoting immunogenic cell death (ICD) and modulating the TME to enhance immune system stimulation. Tissue heating is controlled by both the quantity of GNRs in tissue and the intensity and duration of infrared light exposure. Here, the synergistic potential of GNR‐mediated THT with immunotherapies was explored in immunogenically “cold” mouse tumor models to achieve durable anti‐tumor immunity (Kennedy et al. 2024).

Two mouse models were evaluated for the ability of THT to stimulate the immune system: BALB/c mice bearing 4T1 breast tumors and C57BL/6 mice with B16‐F10 melanoma. After tumors developed, GNRs were intratumorally injected and activated using NIR light to induce sub‐ablative hyperthermia (42°C–48°C) for 5 min. THT reduced tumor burden through cell death mechanisms, including upregulated ICD marked by calreticulin exposure within 48 h; however, tumor regrowth was observed within 6 days post‐treatment. To enhance THT's immunogenic effects, the therapy was combined with i.t. IL‐2. This combination induced robust CD8+ T cell infiltration and led to durable tumor regression in both treated and distant, untreated tumors, as well as the emergence of memory T cells. Additionally, PD‐1 expression, which was upregulated in CD8+ T cells by THT, was targeted with systemic PD‐1 inhibition, further augmenting immune engagement within the TME.

Collectively, these data demonstrated that GNR‐mediated THT effectively initiates a cascade of responses that reduce tumor burden and modulate the TME, potentiating systemic immunity and enhancing the effectiveness of complementary immunotherapies.

#### 2.4.4 Scalable Fabrication of Drug‐Loaded Polymeric Micelles Using Low‐Molecular‐Weight Polyethylene Glycol

Traditional methods for producing drug‐loaded polymeric micelles typically involve dissolving the drug and block copolymer in a non‐selective organic solvent, followed by increasing the medium's selectivity for the shell‐forming block by adding or replacing the solvent with water (Chaibundit et al. 2007; Feng et al. 2020; Fournier et al. 2004; Gaucher et al. 2005; Jette et al. 2004; Kohori et al. 2002; Lavasanifar et al. 2001; Lin et al. 2019; Ma et al. 2015; Tam et al. 2016; Tam et al. 2018; Zhang et al. 2012). However, these approaches face challenges in scalability due to limitations in processing speed, the need for specialty equipment, and incomplete removal of the organic solvent (Desai 2012; Feng et al. 2019; Grodowska and Parczewski 2010; Impurities: Guideline for Residual Solvents Q3C(R*8*) 2021; Payyappilly et al. 2015). To address these issues, Kwon (University of Wisconsin‐Madison) recently developed two innovative methods for fabricating drug‐loaded polymeric micelles using PEG oligomers as the solvent in lieu of conventional small‐molecule solvents. Both methods were successfully applied to PEG_4kDa_‐*b*‐PLA_2.2kDa_ as the model block copolymer and paclitaxel (PTX) or its oligolactic acid prodrug (o(LA)_8_‐PTX) as model drugs.

**FIGURE 2:**
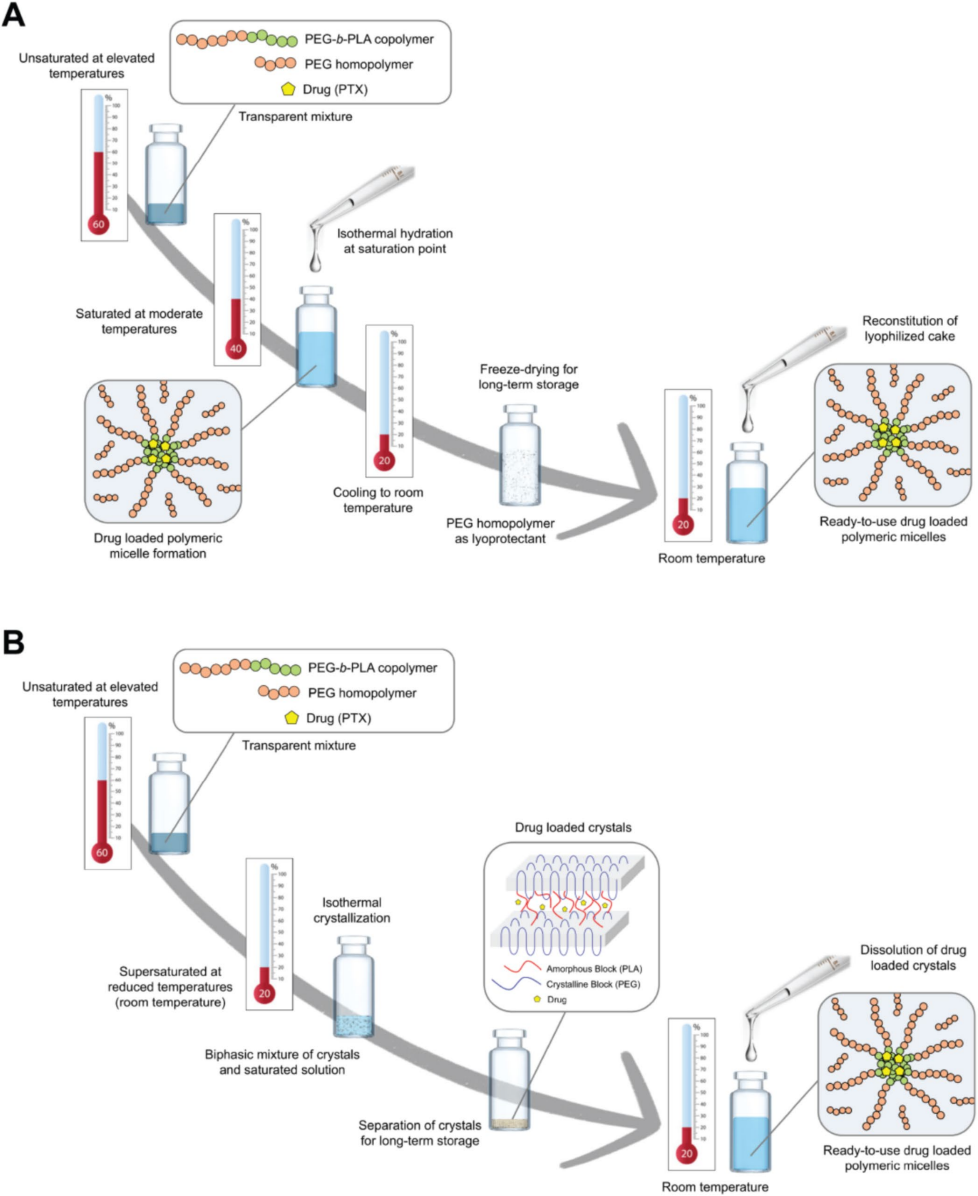
Schematic illustrations of the (A) PEG‐assist method and (B) crystallization of supersaturated solution method for production of drug‐loaded polymeric micelles.

In the first approach, termed the “PEG‐assist” method (Figure 2A), a transparent mixture of PEG oligomer, block copolymer, and drug is formed at elevated temperatures. This mixture is then cooled to its saturation temperature, after which water is added to form drug‐loaded micelles. The resulting aqueous micelle solution can be freeze‐dried without the need for additional lyoprotectants, as the PEG oligomer serves a dual function—acting as a non‐selective solvent during micelle formation and as a lyoprotectant during lyophilization. For this method, PEG oligomers with a molecular weight of 1 kDa or higher are required. For example, encapsulating PTX in PEG_4kDa_‐*b*‐PLA_2.2kDa_ requires PEG_1kDa_ and hydration at 40°C, while encapsulating o(LA)_8_‐PTX requires PEG_2kDa_ and hydration at 60°C.

With the second method, “crystallization of supersaturated solution” (Figure 2B), the mixture undergoes isothermal crystallization at reduced temperatures (e.g., room temperature) instead of hydration at the saturation point. This approach yields semi‐crystalline solids composed of the block copolymer, and the drug and can be stored long‐term in a stable solid form, eliminating the need for lyophilization. Upon hydration, these solids form drug‐loaded micelles. Notably, PEG with molecular weights as low as 200 Da is sufficient for producing PEG_4kDa_‐*b*‐PLA_2.2kDa_ micelles encapsulating PTX or o(LA)_8_‐PTX via this method.

The PEG‐assist and crystallization of supersaturated solution methods share several advantages, including the absence of toxic organic solvents, simplicity of heating–cooling steps, and thermodynamic reproducibility. These features render both methods highly scalable and compliant with Good Manufacturing Practices (GMP). However, there are key differences between the two approaches which influence their suitability for specific applications. The choice of PEG molecular weight is a primary distinguishing factor. The PEG‐assist method typically requires higher molecular weight PEGs, as they serve dual roles—acting as non‐selective solvents during micelle formation and as lyoprotectants during freeze‐drying. Higher molecular weight PEG leads to a higher eutectic temperature of the PEG‐water solution, making freeze‐drying more feasible and efficient. In contrast, the crystallization method does not require freeze‐drying for long‐term storage, as it directly produces semi‐crystalline solids. This eliminates the need for additional lyoprotectant agents and represents a significant advantage over the PEG‐assist method in terms of simplicity and cost‐efficiency. Another notable difference lies in the residual PEG content in the final product. The PEG‐assist method retains nearly all the PEG used during micelle preparation in the final formulation, whereas the crystallization method results in only trace amounts of residual low molecular weight PEG in the separated crystals. Encapsulation capacity also varies between the two methods. The PEG‐assist method achieves nearly complete drug encapsulation within polymeric micelles, making it suitable for applications requiring maximal loading efficiency. In contrast, the encapsulation efficiency in the crystallization method is influenced by the initial copolymer concentration and the quantity of crystals formed. As a result, drug encapsulation capacity is generally lower in the crystallization method.

#### 2.4.5 Normalization of the Tumor Microenvironment to Enable Immune Checkpoint Inhibitors

The widely accepted concept of nanomedicine's enhanced permeability and retention (EPR) effect assumes that anticancer drugs and biologicals can be delivered selectively to tumors due to leaky neovasculature. However, while side effects have been significantly reduced using nanomedicines, improvements in patients' survival have only been modest. By contrast, immune‐checkpoint inhibition (ICI) has provided substantial improvements in the survival of a subset of patients. Unfortunately, however, ICI is estimated to benefit only < 13% of patients. These above findings may be related to the nature of TME. Modulating (“normalizing”) the TME may, therefore, improve nanodrug and ICI distribution into the tumor tissue, thereby improving anticancer therapeutic efficacy. The target cells for TME normalization are cancer‐associated fibroblasts (CAF), which are responsible for the remodeling of the extracellular matrix (ECM) required to develop the TME. TME influences angiogenesis and tumor mechanics as well as modulating the immune system. Transforming growth factor β (TGFβ) is one of the inducers of this conversion of normal fibroblasts to CAFs. Angiotensin receptor blockers (ARB), which are used routinely to reduce high systemic blood pressure, are known to manipulate the TME in part through their inhibition of TGFβ, thereby reprogramming CAF to reduce ECM levels and affect tumor immunity (Martin et al. 2020; Perini et al. 2020; Sahai et al. 2020).

Mouse studies with candesartan (one of the most potent ARB), administered as the free drug, showed the efficacy of candesartan in tumors (Alhusban et al. 2014; Cai et al. 2021; Zhu et al. 2022). In humans, data from a series of retrospective studies involving patients with different cancer types, as well as a prospective phase 2 trial involving patients with locally advanced pancreatic ductal adenocarcinoma, showed that ARB use has the potential to extend patients' survival (Alhusban et al. 2014; Cai et al. 2021; Martin et al. 2020; Murphy et al. 2019; Perini et al. 2020; Sahai et al. 2020; Zhu et al. 2022). However, ARBs cause systemic adverse effects related to the dangerous lowering of blood pressure, preventing their routine use for the treatment of cancer patients. Candesartan delivered via PEGylated small unilamellar liposomes, however, may enable the clinical use of candesartan under conditions that overcome the issue of systemic blood pressure reduction.

The design of such liposomes was based on the extensive experience gained from the development and clinical use of Doxil (Barenholz 2012). The nano‐candesartan used the same lipid composition as Doxil. Further, to achieve an effective candesartan level in the tumor, the nano‐liposomes were similar in size to Doxil, affording benefit from the EPR effect and a long circulation time. Finally, to avoid the reduction in blood pressure, candesartan was stably loaded, minimizing free drug in the formulation and allowing almost no release in plasma. For candesartan, which is an amphipathic weak acid, the remote active loading is driven by a transmembrane acetate gradient and supported by the intra‐liposome calcium ions and hydroxy‐propyl beta‐cyclodextrin (HPCD). The resulting nano‐candesartan demonstrated no release in serum in vitro and almost none in the circulation; it also did not affect mice systemic mean blood pressure. In vitro studies revealed release of candesartan from the liposomes in the presence of tumors due to tumor metabolites. Pharmacokinetic (PK) and biodistribution studies demonstrated prolonged circulation time and accumulation at the tumor site. Finally, efficacy studies in a mouse 4T1 model showed that nano‐candesartan inhibited, by itself, to some extent, tumor growth. Most importantly, it dramatically improved the activity of ICI in this model (in which ICI by itself was effectless). Immunohistochemistry of the tumors confirmed that nano‐candesartan significantly reduced α‐smooth muscle actin (αSMA, a CAF biomarker) and collagen 1, suggesting normalization of the TME, which explains the improved therapeutic efficacy of the nano‐candesartan/ICI combination in this mouse tumor model.

## 3 Evolution of the Field: A Perspective From the Nanotechnology Characterization Laboratory

**FIGURE 3:**
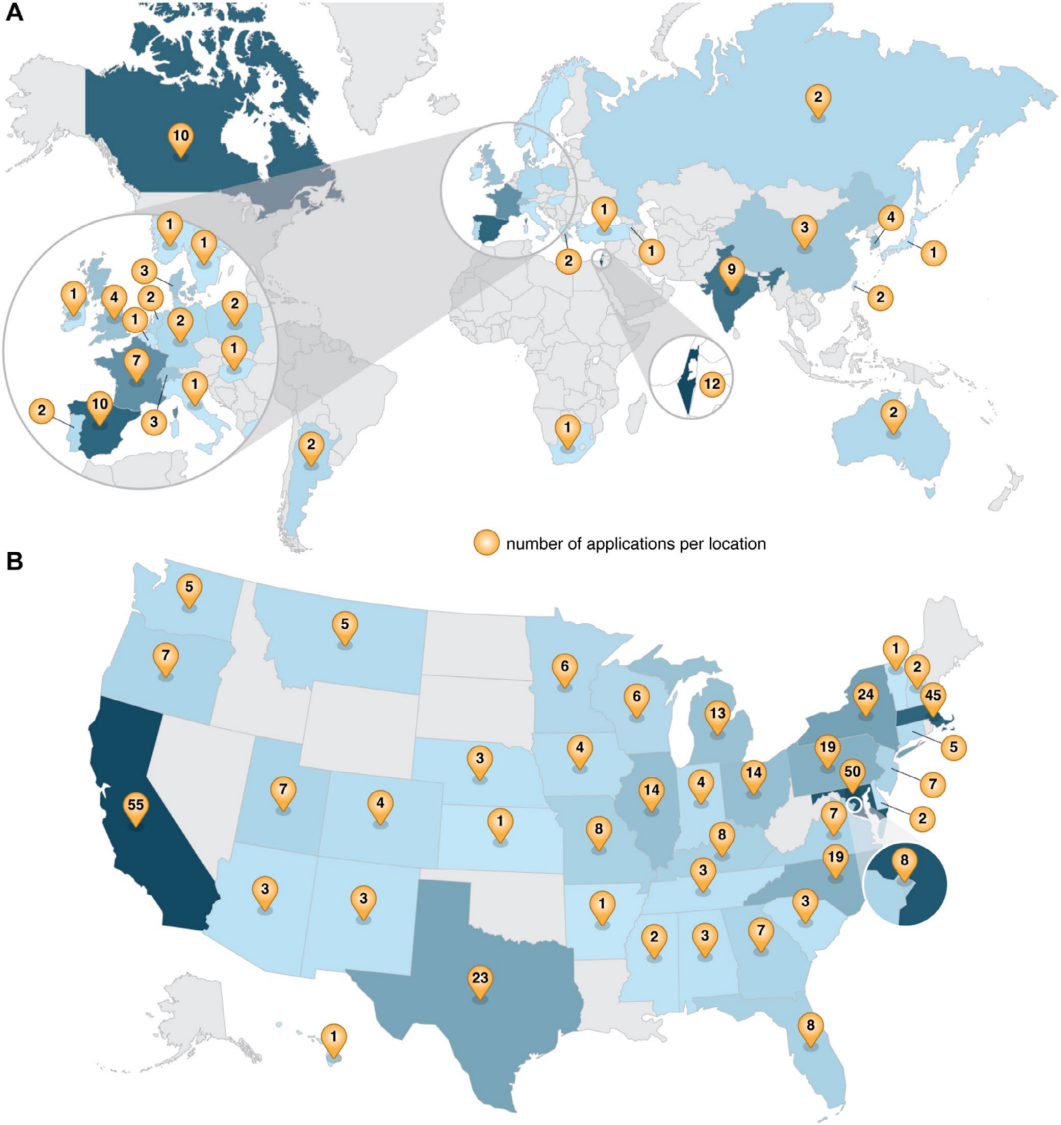
Global interest in the NCL's Assay Cascade program. (A) A global heat map shows the international applicants to the program, with applications from 30 different countries from six continents. (The U.S. data were not included in this heat map to allow better visualization of the other countries). (B) A heat map of the United States highlights where applications to the program originated, with applications from 39 states and the District of Columbia. The yellow pins indicate the number of applications from each country or state.

Over the last two decades, the NCL has had an intimate look at the progress and advancements of cancer nanotechnology—thanks entirely to the hundreds of collaborations established around the world. The unique, multidisciplinary nature of the NCL program has attracted nanomedicine researchers and developers from around the globe, having received applications from 30 different countries across six continents (Figure 3A). Within the United States, applications have come from 39 states and the District of Columbia (Figure 3B).

**FIGURE 4:**
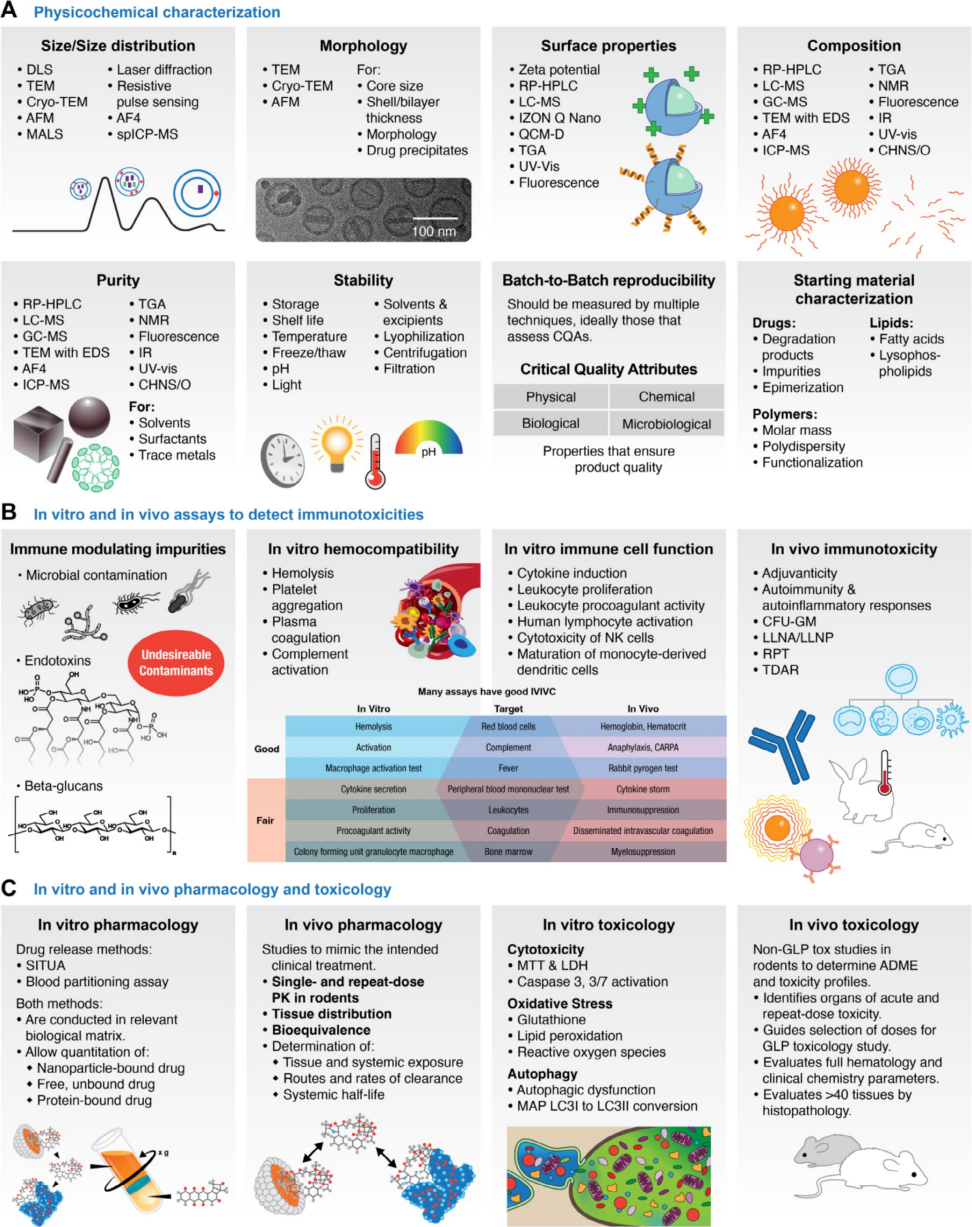
NCL's Assay Cascade. (A) Physicochemical characterization of nanomaterials is intended to support the Chemistry, Manufacturing, and Controls (CMC) section of an Investigational New Drug (IND) application and typically includes tests for the eight indicated properties. Common techniques used for each are included. (B) Immunological characterization is intended to identify potential immunotoxicity concerns in the preclinical stage—one of the most common reasons for clinical failure. Assays include the detection and quantitation of innate immune response modulating impurities (IIRMI), in vitro hemocompatibility tests, in vitro assays to assess effects on the function of immune components, and various in vivo immunotoxicity protocols. The in vitro–in vivo correlation table was adapted from (Dobrovolskaia and McNeil 2013b). (C) Pharmacokinetic and toxicity testing includes in vitro and in vivo assays designed to inform future clinical trials, identifying tissue and systemic exposure, routes and rates of clearance, systemic half‐life, and establishing potential organs of toxicity. More details about the NCL Assay Cascade, including full‐text protocols, are available on the NCL website at https://www.cancer.gov/nano/research/ncl/protocols‐capabilities. ADME, absorption, distribution, metabolism, excretion; AF4, asymmetric‐flow field‐flow fractionation; AFM, atomic force microscopy; CFU‐GM, colony‐forming unit‐granulocyte‐macrophage; CHNS/O, carbon, hydrogen, nitrogen, sulfur, oxygen elemental analyzer; cryo‐TEM, cryogenic transmission electron microscopy; CQA, critical quality attribute; DLS, dynamic light scattering; EDS, energy dispersive x‐ray spectroscopy; GC–MS, gas chromatography–mass spectrometry; GLP, good laboratory practices; IR, infrared; IVIVC, in vitro–in vivo correlation; LC–MS, liquid chromatography‐mass spectrometry; LDH, lactate dehydrogenase membrane integrity assay; LLNA, local lymph node assay; LLNP, local lymph node proliferation assay; MALS, multi‐angle light scattering; MAP LC3, microtubule‐associated protein light chain 3; MTT, 3‐[4,5‐dimethylthiazol‐2‐yl]‐2,5 diphenyl tetrazolium bromide cell viability assay; NK, natural killer; NMR, nuclear magnetic resonance; QCM‐D, quartz crystal microbalance with dissipation; RP‐HPLC, reversed‐phase high‐performance liquid chromatography; RPT, rabbit pyrogen test; SITUA, stable isotope tracer ultrafiltration assay; spICP‐MS, single particle inductively coupled plasma mass spectrometry; TDAR, T cell dependent antibody response; TEM, transmission electron microscopy; TGA, thermogravimetric analysis; UV–vis, ultraviolet–visible.

**FIGURE 5:**
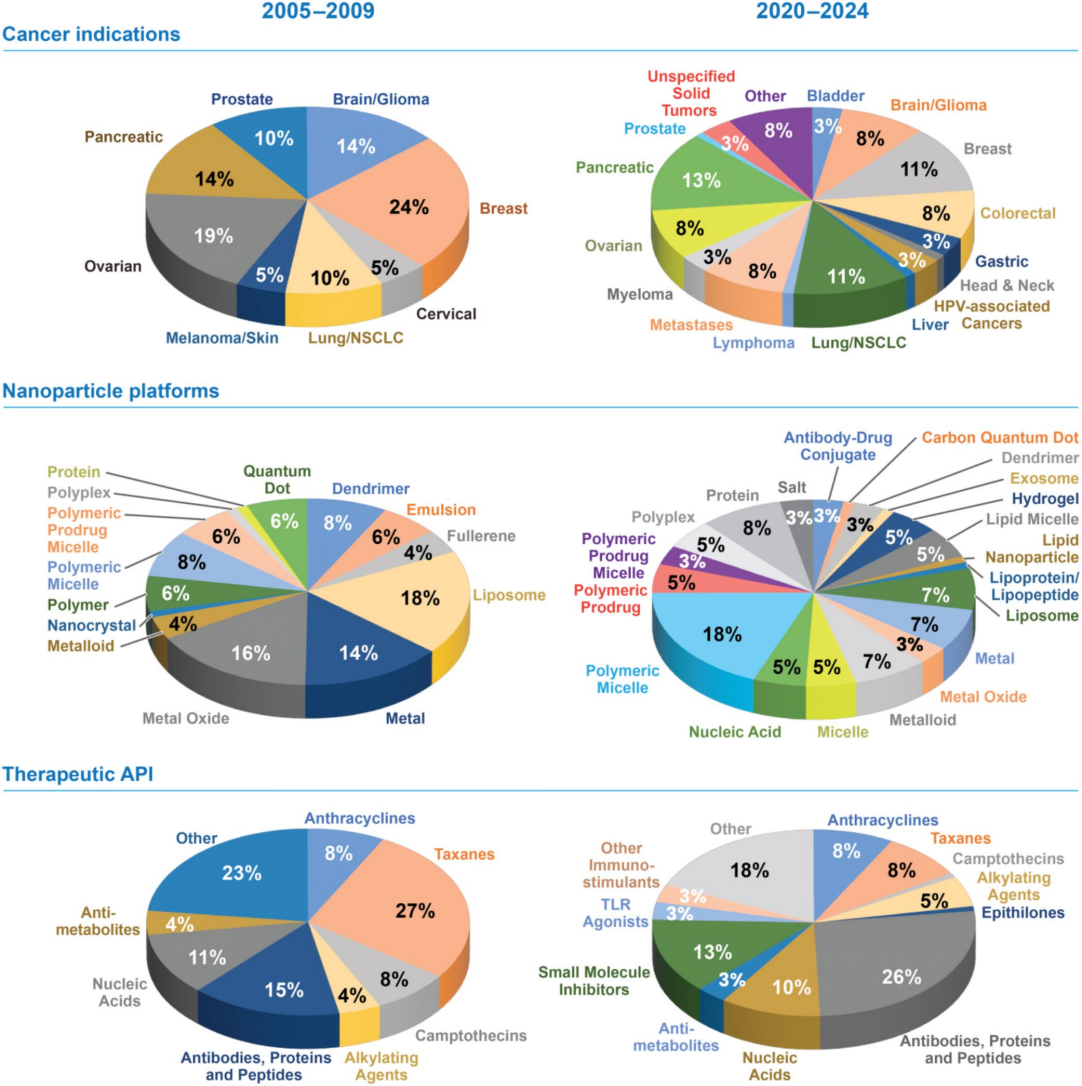
Trends in nanoparticle submissions to the NCL's Assay Cascade characterization program between 2005–2009 and 2020–2024 showing the broadening of cancer indications studied, nanoparticle platforms utilized, and therapeutic active pharmaceutical ingredients (API) incorporated. Wedges without a value are ≤ 2% 2024.

NCL characterization of nanomaterials progresses through what is termed the “Assay Cascade,” a series of physicochemical analyses and both in vitro and in vivo studies in immunology, toxicology, and pharmacology designed to thoroughly characterize the physical, chemical, and biological properties of a material to inform its translational development (Figure 4). The Assay Cascade, first implemented in 2005, continually evolves to keep pace with the changing landscape of nanomedicine research. Early concepts seen by the program were primarily liposome (18%) and metallic‐based formulations (30%), taxanes were the predominant active pharmaceutical ingredient (API) studied (27%), and cancer indications focused on breast (24%), ovarian (19%), pancreatic (14%), and brain (glioma; 14%) cancers (Figure 5) (Nanotechnology Characterization Laboratory 2024). Fast‐forward 20 years and the field has diversified in all areas. Cancer indications are no longer focused just on the most commonly diagnosed and/or notoriously difficult‐to‐treat cancers. More than a dozen different cancers were included in concepts submitted from the last 5 years, including rare cancers and those specifically targeting metastases. Polymeric/polymeric prodrug micelles are now the most popular platform (> 20%), while liposomes (7%), and metallic‐based (10%) formulations both decreased in usage but were still viable preclinical candidates. Finally, biological entities such as antibodies, proteins, peptides, and various nucleic acids have overtaken traditional cytotoxic small molecules as the most prevalent therapeutic API studied, in large part due to increased exploration of immunotherapy/vaccine and personalized treatment approaches and improved delivery vehicles such as LNP.

**FIGURE 6:**
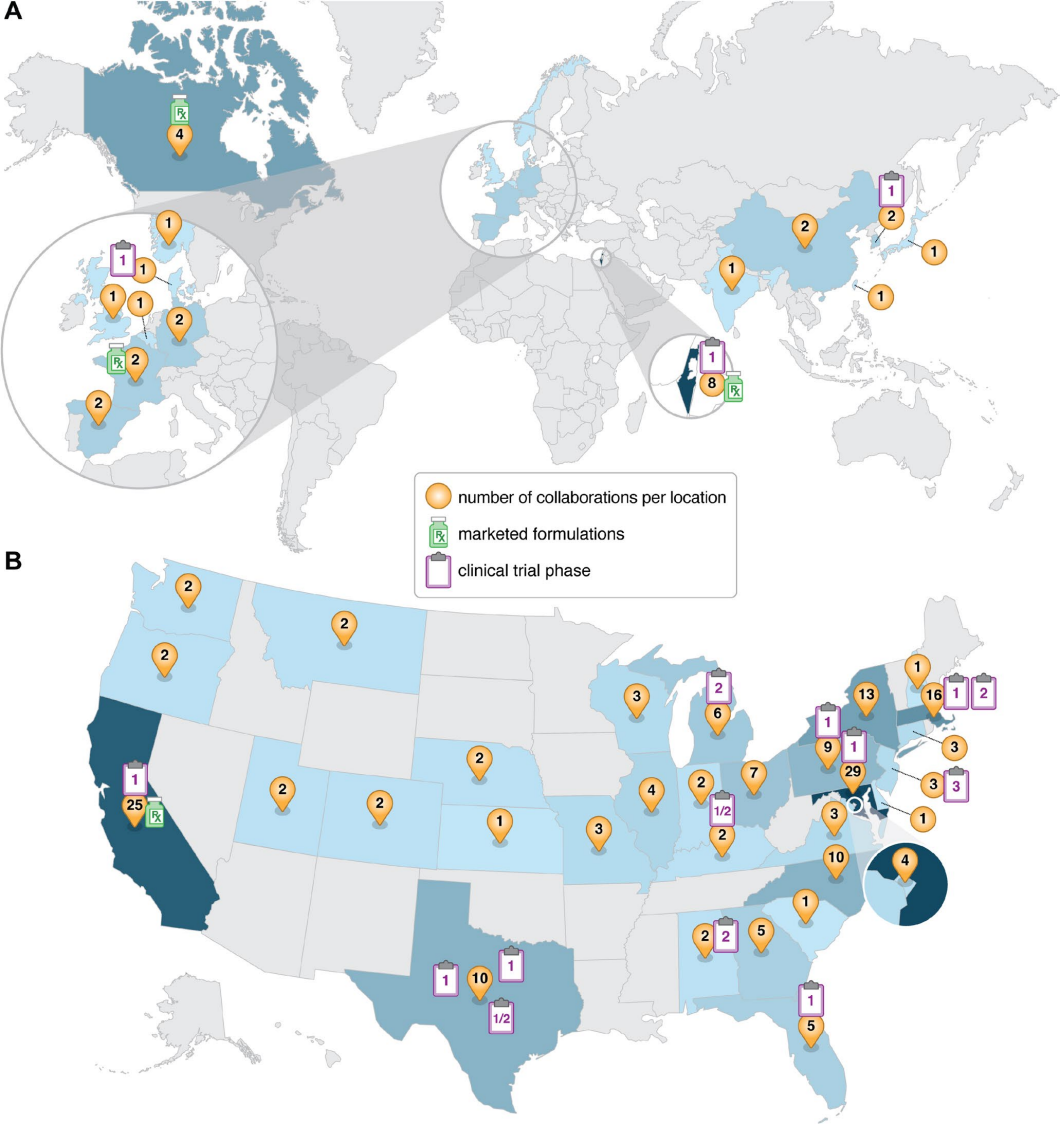
NCL's Assay Cascade collaborations and clinical success. (A) A global heat map shows the international collaborations accepted into the program, with projects originating from 14 different countries. (The U.S. data were not included in this heat map to allow better visualization of the other countries.) Among the global collaborations, three nanomedicines have advanced to phase 1 clinical trials (from Denmark, Israel, and South Korea), and three nanomedicines are now marketed (from Canada, France, and Israel). (B) A heat map of the United States highlights the collaborations accepted into the program, with projects from 30 states and the District of Columbia. Of the national collaborations, 14 nanomedicines have advanced to human clinical trials, spanning from early phase 1 up to phase 3, and one nanomedicine is now marketed. The yellow pins indicate the number of collaborations from each country or state. The clipboard icon represents a nanoformulation that advanced to clinical trials, with the inset number indicating the clinical trial phase. The bottle icon denotes a marketed formulation originating from that location.

This work has seen nearly two dozen novel cancer nanomedicine concepts reach human clinical trials, several of which are now approved and actively used to benefit cancer patients. Globally, NCL collaborators in Canada, France, and Israel now have commercially marketed nanoformulations in the United States and/or Europe, while NCL collaborators from Denmark, Israel, and South Korea have advanced their novel concepts to phase 1 clinical trials (Figure 6A). Among U.S. collaborations, 14 novel nanomedicines have reached clinical trials, and one is now marketed—Fyarro (Figure 6B). In addition, the NCL has also witnessed tremendous advances in characterization technology and instrumentation, seen the commercialization of generic and follow‐on nanomedicines, and observed development of novel, next‐generation nanoparticles.

### 3.1 Physicochemical Characterization

When the NCL first began characterizing nanoparticles as part of the Assay Cascade program, there were only three analytical instruments in the lab—a dynamic light scattering (DLS) instrument with zeta potential capabilities, a reversed‐phase high‐performance liquid chromatography (RP‐HPLC) stack with UV–vis and fluorescence detectors, and an asymmetric‐flow field‐flow fractionation (AF4) instrument. Significant efforts were made to study the nuances of DLS and zeta potential measurements for various nanoparticle samples, including sample preparation, reporting size, multiple scattering, viscosity, absorbance, rotational diffusion, and resolving power, and to develop protocols that could be widely adapted across this diverse research space (Caputo et al. 2019; Clogston 2021; Clogston et al. 2019; Clogston and Patri 2011, 2013; Clogston and Vermilya 2020; Hackley and Clogston 2010, 2011; Smith et al. 2017). These techniques are fundamental to nanomaterials and were later adapted as part of the “NCL prescreen,” that is, tests conducted prior to any other analyses, to ensure the integrity of the nanomaterials (Crist et al. 2013). RP‐HPLC quickly evolved from simple UV–vis and/or fluorescence detection of total drug to include the use of centrifugal filtration devices to afford separation of free drug for quantitation of the free/non‐nanoparticle associated drug fraction—a useful measurement for evaluating formulation encapsulation efficiency, nanoparticle stability, and lot‐to‐lot variations. Incorporation of charged aerosol detection (CAD) allowed for broader detection capabilities of not only other APIs but also measurement of individual lipid concentrations, lipid impurities, and degradation products (i.e., free fatty acids and lysophospholipids due to hydrolysis), specialized ion concentrations (i.e., used for active drug loading), buffer components, and excipients (Wu et al. 2019; Xu and Clogston 2024). By further expanding detection capabilities to include mass spectrometry, cholesterol oxidation products (oxysterols) and other component impurities and degradation products could also be readily identified. To date, NCL has developed sample preparation and RP‐HPLC methods for over 40 different APIs, from small molecules to proteins, peptides, and nucleic acids (Table 1). Likewise, AF4 has also seen tremendous growth in utility. In addition to simple size distribution (flow‐mode DLS), size/size distribution can be measured in the presence of human plasma to afford a qualitative assessment of protein binding to the nanoparticle surface. Furthermore, this separation technique allows for the collection of fractions (based on size) that can be analyzed off‐line by any number of other analytical techniques (RP‐HPLC, inductively coupled plasma mass spectrometry [ICP‐MS], cryogenic transmission electron microscopy [cryo‐TEM], etc.), thus making AF4 an extremely powerful characterization technique for greater insight into drug loading as a function of nanoparticle size, nanoparticle stability and drug partitioning, and batch‐to‐batch consistency (Caputo et al. 2019; Caputo et al. 2021; Clogston and Hu 2020; Hansen and Clogston 2024a, 2024c; Hu et al. 2020).

**TABLE 1:** Summary of API measured by RP‐HPLC. The table provides a summary of the column, detector, and mobile phase suitable for RP‐HPLC detection of various APIs. Importantly, these may be influenced by the specific nanoparticle and required sample preparation procedure.

Active pharmaceutical ingredient	Column	Detector	Mobile phase
2‐deoxy‐D‐glucose	SIELC Primesep S2	CAD	A = Water w/0.1% (vol/vol) TFA
			B = Acetonitrile w/0.1% (vol/vol) TFA
Alendronate	C18	CAD	A = 18 mM amylamine, pH 7 (adjusted with acetic acid)
			B = Acetonitrile
Amphotericin B	C18	UV	A = Water w/0.1% (vol/vol) TFA
			B = Acetonitrile w/0.1% (vol/vol) TFA
Bortezomib	C18	UV	A = Water w/0.1% (vol/vol) TFA
			B = Acetonitrile w/0.1% (vol/vol) TFA
Brefeldin A	C18	UV	A = Water w/0.1% (vol/vol) TFA
			B = Methanol w/0.1% (vol/vol) TFA
Breflate	C18	UV	A = Water w/0.1% (vol/vol) TFA
			B = Methanol w/0.1% (vol/vol) TFA
Cabazitaxel	C18	UV	A = Water w/0.1% (vol/vol) TFA
			B = Acetonitrile w/0.1% (vol/vol) TFA
Camptothecin/prodrug	C18	UV	A = Water w/0.1% (vol/vol) TFA
			B = Acetonitrile w/0.1% (vol/vol) TFA
Cisplatin/prodrug	C18	UV	A = Water w/0.1% (vol/vol) TFA
			B = Acetonitrile w/0.1% (vol/vol) TFA
Daunorubicin	C18	UV, FL	A = Water w/0.1% (vol/vol) TFA
			B = Acetonitrile w/0.1% (vol/vol) TFA
Docetaxel	C18	UV	A = Water w/0.1% (vol/vol) TFA
			B = Acetonitrile w/0.1% (vol/vol) TFA
Doxorubicin/prodrug	C18	UV, FL	A = Water w/0.1% (vol/vol) TFA
			B = Acetonitrile w/0.1% (vol/vol) TFA
Echinomycin	C18	UV	A = Water w/0.1% (vol/vol) TFA
			B = Acetonitrile w/0.1% (vol/vol) TFA
Epirubicin	C18	UV, FL	A = Water w/0.1% (vol/vol) TFA
			B = Acetonitrile w/0.1% (vol/vol) TFA
Epothilone D	C18	UV	A = Water w/0.1% (vol/vol) TFA
			B = Methanol w/0.1% (vol/vol) TFA
Folate/folic acid	C18	UV, FL	A = Water w/0.1% (vol/vol) TFA
			B = Acetonitrile w/0.1% (vol/vol) TFA
α‐galactosyl ceramide	C8	CAD, MS	A = Water w/0.1% (vol/vol) TFA
			B = EtOH:MeOH (70:30 by volume), 0.5% formic acid, 10 mM ammonium formate
Gemcitabine/prodrug	C18	UV	A = Water w/0.1% (vol/vol) TFA
			B = Acetonitrile w/0.1% (vol/vol) TFA
Glycine	C18	UV, CAD	A = Water w/0.1% (vol/vol) TFA
			B = Acetonitrile w/0.1% (vol/vol) TFA
Irinotecan/prodrug	C18	UV	A = Water w/0.1% (vol/vol) TFA
			B = Acetonitrile w/0.1% (vol/vol) TFA
Ixabepilone	C18	UV	A = 90% 5 mM Tris–HCl (pH 8.0), 10%ACN
			B = 90%ACN, 10% 5 mM Tris–HCl (pH 8.0)
Melittin	C18	UV	A = Water w/0.1% (vol/vol) TFA
			B = Acetonitrile w/0.1% (vol/vol) TFA
Metformin	C8	UV, MS	A = Water w/0.1% (vol/vol) TFA
			B = Acetonitrile w/0.1%(vol/vol) TFA
Methotrexate	C18	UV	A = Water w/0.1% (vol/vol) TFA
			B = Acetonitrile w/0.1% (vol/vol) TFA
Mupirocin	C18	UV	A = Water w/0.1% (vol/vol) TFA
			B = Methanol w/0.1% (vol/vol) TFA
Paclitaxel	C18	UV	A = Water w/0.1% (vol/vol) TFA
			B = Acetonitrile w/0.1% (vol/vol) TFA
Prednisolone phosphate	C18	UV	A = Water w/0.1% (vol/vol) TFA
			B = Acetonitrile w/0.1% (vol/vol) TFA
Propofol	C18	UV	A = Water w/0.1% (vol/vol) TFA
			B = Acetonitrile w/0.1% (vol/vol) TFA
Quinine	C18	UV	A = Water w/0.1% (vol/vol) TFA
			B = Methanol w/0.1% (vol/vol) TFA
Rapamycin	C18	UV	A = Water w/0.1% (vol/vol) TFA
			B = Methanol w/0.1% (vol/vol) TFA
Simvastatin	C18	UV	A = Water w/0.1% (vol/vol) TFA
			B = Acetonitrile w/0.1% (vol/vol) TFA
SN‐38/prodrug	C18	UV	A = Water w/0.1% (vol/vol) TFA
			B = Methanol w/0.1% (vol/vol) TFA
Telratolimod	C18	UV	A = Water w/0.1% (vol/vol) TFA
			B = Acetonitrile w/0.1% (vol/vol) TFA
Trastuzumab	C8	UV, FL	A = Water w/0.1% (vol/vol) TFA
			B = iPrOH/ACN/H_2_O/TFA (70/20/9.9/0.1 by volume)

Abbreviations: ACN, acetonitrile; CAD, charged aerosol detector; EtOH, ethanol; FL, fluorescence detector; iPrOH, isopropanol; MeOH, methanol; MS, mass spectrometer detector; TFA, trifluoroacetic acid; UV, ultraviolet–visible detector; vol/vol, volume‐to‐volume ratio.

These three instruments—DLS, RP‐HPLC, and AF4—can provide a wealth of data, including measurement of size by multiple techniques, zeta potential, full compositional analysis to include total, bound, and free drug, purity assessment, drug release, stability, and lot‐to‐lot consistency. As new instrumentation was acquired, additional techniques afforded even more data. Headspace gas chromatography was later added to the Assay Cascade to measure residual organic solvents (Kattel and Clogston 2022, 2023, 2024). Nanoparticle concentration can now be measured by resistive pulse sensing (Caputo et al. 2019; Vermilya and Clogston 2024), light scattering (Caputo et al. 2019), and, for metallic nanoparticles, single particle inductively coupled plasma mass spectrometry (spICP‐MS) (Hansen and Clogston 2021, 2024b). ICP‐MS can also be used for metal quantitation of not only metallic nanoparticle stock samples but also for blood and tissue distribution studies (Yu et al. 2010a, 2010b), as well as determination of residual metallic impurities used during formulation. Today's physicochemical characterization Assay Cascade includes these as well as other techniques to afford a comprehensive physical and chemical analysis of the formulation suitable to address many of the requirements in the Chemistry, Manufacturing, and Controls (CMC) section of an Investigational New Drug (IND) portfolio (Figure 4A). A summary of the parameters, methods, and considerations for the physicochemical characterization of the three most common nanoparticle platforms—lipid‐based, polymer‐based, and metallic‐based—has also been published to serve as a guide for researchers, highlighting the advancements of physicochemical characterization over the last 20 years (Clogston 2024a, 2024b, 2024c).

### 3.2 Immunology

Traditionally, immunotoxicity assessment involves the analysis of two major types of adverse effects: immunosuppression and immunostimulation. The third common type of immunotoxicity commonly seen with nanomaterials includes immunomodulation. While many nanotechnology platforms are not overtly immunosuppressive or immunostimulatory themselves, they change the way the immune system responds to otherwise immunostimulatory or immunosuppressive substances. Nanoparticles often have subtle or even mixed effects, which makes this toxicity more challenging to discover during the preclinical phase. Oxidative stress, lysosomal dysfunction, mitochondrial stress, and changes in cellular respiration are common mechanisms of immunotoxicity of drug‐free nanotechnology carriers (Hamilton et al. 2009; Ilinskaya et al. 2015; Shah et al. 2018; Yuan et al. 2019; Zhang et al. 2012). The NCL immunology Assay Cascade (Figure 4B), first launched in 2005, utilized existing traditional in vitro and in vivo immunotoxicity methods optimized for nanoparticle characterization. The main optimization steps, consistent across all assays, involved (1) scaling down volume requirements due to the limited quantities of nanomaterials available for preclinical studies and (2) overcoming a broad spectrum of interferences due to nanoparticle physicochemical properties (e.g., intrinsic fluorescence or absorbance at the assay wavelength, cationic charge, large surface area) or function (e.g., fluorescence quenching, protein binding, enzymatic activity).

Application of the Assay Cascade in characterization of the diverse portfolio of nanomaterials submitted to the program revealed several common challenges and nanoparticle class‐specific properties. For example, ~5% and 30% of nanoparticles annually fail the first stage (NCL prescreen) due to bacterial and endotoxin contamination, respectively. In both cases, common sources of contamination include water, dust, handling, and processing. Bacterial strains in the contaminated nanoparticle samples commonly include aquatic and soil species as well as pathogenic and opportunistic human microflora (Table 2). Excessive amounts of endotoxin in nanoformulations confound the results of efficacy and toxicity studies, cause undesirable toxicity, contribute to the immunogenicity of protein‐based API or targeting ligands, and exaggerate endotoxin‐mediated inflammation through a variety of mechanisms, including proton sponge effect, lysosomal rupture, and inactivation of negative regulators of inflammation (Dobrovolskaia 2017; Dobrovolskaia, Patri, Potter, et al. 2012; Ilinskaya et al. 2014). This emphasizes the importance of using pyrogen‐free materials and supplies as well as depyrogenating equipment used for nanoparticle synthesis. Common tips for reducing and eliminating endotoxin contamination from nanoparticles, along with approaches for overcoming assay interferences, have been described elsewhere (Dobrovolskaia 2023; Dobrovolskaia et al. 2010; Dobrovolskaia et al. 2014; Neun and Dobrovolskaia 2024). While nanoparticle physicochemical properties such as size, charge, and surface functionalities determine their interactions with the immune system (Avila et al. 2021; Dobrovolskaia 2017; Dobrovolskaia and McNeil 2007; Dobrovolskaia, Patri, Simak, et al. 2012; Enciso et al. 2016; Grunberger, Dobrovolskaia, et al. 2024; Grunberger, Newton, et al. 2024; Hong et al. 2018; Ilinskaya et al. 2019; Newton, Radwan, et al. 2023; Newton, Zhang, et al. 2023), the NCL Assay Cascade revealed two remarkable properties common for all polymer‐ and lipid‐containing formulations: (1) prolongation of plasma coagulation time (especially in the activated partial thromboplastin time [APTT] pathway), and (2) exclusive induction of chemokines—such as IL‐8, MCP‐1, MCP‐2, MIP‐1α, MIP‐1β, and RANTES—in the absence of other proinflammatory cytokines and interferons (Dobrovolskaia 2022).

**TABLE 2:** Commonly identified bacterial strains in preclinical nanoformulations.

Bacterial strain	Common source
*Achromobacter marplatensis*	Soil
*Burkholderia cenocepacia*	Soil, water
*Burkholderia cepacian*	Soil, water
*Burkholderia contaminans*	Soil, water
*Burkholderia metallica*	Soil, water
*Caulobacter segnis*	Soil
*Citrobacter freundii*	Soil, water, food, human intestinal tract
*Leifsonia lichenia*	Lichen
*Ochrobactrum anthropic*	Soil, water, plants, healthcare environments
*Phreatobacter oligotrophus*	Ultrapure water
*Pseudomonas beteli*	Soil, water, plants
*Ralstonia pickettii*	Soil, water, biofilms on plastic
*Rhizobium halotolerans*	Soil
*Rhodococcus baikonurensis*	Soil
*Rothia terrae*	Soil
*Sphingomonas aeria*	Soil, water, healthcare environments
*Sphingomonas zeae*	Internal stem tissue of corn plants
*Staphylococcus haemolyticus*	Human skin

Other completed and ongoing studies leverage structure–activity relationships (Avila et al. 2021; Dobrovolskaia, Patri, Simak, et al. 2012; Enciso et al. 2016; Grunberger, Dobrovolskaia, et al. 2024; Grunberger, Newton, et al. 2024; Hong et al. 2018; Ilinskaya et al. 2019; Newton, Radwan, et al. 2023) to develop quantitative and artificial intelligence models (Chandler et al. 2022; Johnson et al. 2017), expand traditional immunology definitions (e.g., the use of the term phagocytosis in application to nanoparticles) (França et al. 2011), and elaborate on in vitro–in vivo correlations to reduce animal usage (Cedrone et al. 2024; Potter et al. 2024).

### 3.3 Pharmacology and Toxicology

NCL pharmacology and toxicology has had several main themes over the past two decades, focusing on pharmacokinetics and toxicological mechanisms common to nanomedicines (Figure 4C). The primary hurdle for evaluating nanomedicine pharmacokinetics is the need to measure drug fractions, encapsulated and unencapsulated drug, with encapsulated drug acting as a drug depot, and unencapsulated or released drug being the active fraction. The ability to accurately measure nanomedicine drug fractions is very important from a regulatory perspective, as it is a common requirement of bioanalytical methods for in vitro drug release and pharmacokinetic/bioequivalence evaluation found in FDA guidance and EMA reflection documents (Ambardekar and Stern 2015; Drug products, including biological products, that contain nanomaterials. Guidance for industry 2022). The first decade at NCL focused on the use of pharmacokinetic modeling to estimate drug fractions, as general sample preparation methods to separate fractions were not available (Ambardekar and Stern 2015). However, with the NCL's development of the stable isotope tracer ultrafiltration assay (SITUA), a precise and general assay was established to measure nanomedicine drug fractions, no longer needing to rely on indirect methods such as modeling to estimate fractions (Skoczen et al. 2015; Stern et al. 2024). The SITUA method has been very successful in evaluating nanomedicine pharmacokinetics, assisting in formulation optimization and determination of bioequivalence and lot‐to‐lot variability (Hwang et al. 2021; Skoczen et al. 2020).

Changes in drug toxicity profiles resulting from nanomedicine‐mediated alterations in drug distribution have been observed, with doxorubicin liposome‐associated palmar‐plantar erythrodysesthesia (PPE) being a classic example (Lorusso et al. 2007). In addition to changing tissue drug exposure, nanomedicine platforms can have inherent toxicity resulting from their physicochemical properties, often resulting from induction of oxidative stress and inflammation (Stern and McNeil 2008). In addition to these more common mechanisms of toxicity, autophagic dysfunction has also been associated with nanomaterial toxicity, especially for biopersistent nanomaterials that accumulate in lysosomes, and is an active area of nanotoxicology research at the NCL (Stern et al. 2012). Recent studies in the autophagy area have focused on the signaling pathways involved in nanomaterial‐autophagy interaction and toxicological sequelae (Zhou et al. 2024).

While nanomedicines have, in the past, been regulated identically to small molecules and biologics from a toxicological perspective (Drug products, including biological products, that contain nanomaterials. Guidance for industry 2022; Stern et al. 2010), including toxicity evaluation of the drug and non‐drug, excipient components of the formulations separately, this practice has recently been called into question. The idea of evaluating the toxicity of the nanomedicine as a single entity, rather than the drug and individual components separately, is rooted in the idea that tissues are exposed to the intact complex, in specific ratios, and testing the components individually may not only be a waste of resources, but it may also be misleading (Hemmrich and McNeil 2023). Further, tissue exposure itself is governed by the physicochemical properties of the nanomedicine entity, while tissues exposed to the individual components is likely to be very different. This shift in regulatory paradigms toward the “nanomedicine is the drug”, if adopted in the future, would streamline the testing of nanomedicines composed of novel materials.

### 3.4 Nanotechnology Formulation

Cancer nanomedicine formulation has seen changes in underlying dogma over the past 20 years, with the EPR theory of tumor nanoparticle accumulation put forth by Maeda et al. dominating the first decade (Matsumura and Maeda 1986). This EPR theory has since been questioned, with concerns over the lack of corresponding vascular architecture in preclinical and clinical tumors, difficulty in reproducing preclinical results clinically, and controversial findings of low nanoparticle tumor accumulation (Nichols and Bae 2014; Price et al. 2020; Wilhelm et al. 2016). New theories for nanoparticle tumor accumulation have now emerged, with evidence of nanoparticle tumor uptake via active endothelial transport and receptor‐mediated transcytosis as opposed to transport through vascular fenestrations (Doaa et al. 2024; Sindhwani et al. 2020). Correspondingly, nanomedicine active tumor targeting has been revised to incorporate ligands for these vascular transcytosis uptake mechanisms in addition to the tumor cells (Doaa et al. 2024).

The introduction of stimuli responsive nanocarriers is another area of recent inquiry. These responsive drug carriers are engineered to release their cargo upon exposure to external stimuli (such as ultrasound) or internal stimuli (such as the TME or cancer cells), thus minimizing collateral damage to healthy tissue (Mi 2020; Zhao et al. 2021). Coupled with active targeting, these stimuli responsive nanocarrier formulations show great promise in treating cancers that require precise delivery of potent drugs (Abousalman‐Rezvani et al. 2024). The “Trojan horse” concept is an alternative approach for the active delivery of nanoparticles to cancer tissue by loading into natural or engineered immune cells, predominantly macrophages (Ding et al. 2021). Another innovative targeting method utilizes “nanoghosts,” which are part synthetic and part derived from cell membranes and demonstrate selective targeting capabilities for cancer therapy (Krishnamurthy et al. 2016).

Cancer diagnostic nanocarriers is another niche area that has developed dramatically over the past two decades (Dessale et al. 2022; Liu and Grodzinski 2021). Nanocarrier‐based imaging systems have the potential to reduce contrast agent toxicity and improve sensitivity and specificity, crucial attributes that can allow for early detection of cancers. Early detection is of paramount importance for cancer, especially pancreatic cancer where the stage of diagnosis plays a key role in therapeutic outcomes (Singhi et al. 2019). Nanocarrier formulations of NIR fluorophores have resulted in improvements in surgical resection of solid tumors, owing to the unique characteristics of NIR fluorophores, including increased depth of penetration and reduced scattering and autofluorescence (Baghdasaryan et al. 2024; Bortot et al. 2023). Nanocarriers using multiple imaging modalities, such as conventional MRI combined with photoacoustic imaging, allow for precise image‐guided tumor resection (Thawani et al. 2017). Development of nanoparticle imaging agents targeting the immune system has the potential to improve immunotherapy through better patient selection, as well as evaluation of immunomodulatory response (Crist et al. 2021).

A very recent advancement in nanomedicine formulation, with great potential for cancer prophylaxis and therapy, is the development and marketing of various nanoparticle platforms for nucleic acid delivery, highlighted by the success of LNP mRNA vaccines (Miao et al. 2021). The LNP platform also has potential for delivery of other oligonucleotide therapeutics, such as siRNA and CRISPR/Cas9, for silencing of non‐druggable protein targets and gene editing, respectively (El Moukhtari et al. 2023; Kazemian et al. 2022). Encapsulating DNA in the LNP platform may be used as a vaccine adjuvant or to express a protein of interest (Liao et al. 2024). Although LNP liver and lymph node delivery pathways are well understood, selective targeting of other tissues of therapeutic interest is still challenging and an area of active research (Kularatne et al. 2022).

## 4 Prospective on the Future of the Field

The nanomedicine field is dynamic and both reflects on and is affected by the changes in other areas of drug development. Advances in nanotechnology formulation to include multi‐component, multi‐stage, and stimuli‐responsive materials are demanding more from preclinical characterization programs. These changes and increases in the complexity of nanoformulations pose specific challenges for nanoparticle characterization, requiring equally complex advancements in instrumentation and a multifaceted approach to defining not only the physical, chemical, and biological properties of a formulation but also the unique influences each has on the other.

### 4.1 Multi‐Component Delivery Systems

A substantial part of the field of multi‐component (or composite) therapeutic agents—perhaps the majority of them—is an emanation of nanomedicine. “Traditional” (passively targeted) nanomedicines comprise at least two components: the nanoparticle and the active agent comprised within. The addition of surface modification, such as through derivatization with biomolecular recognition moieties (as in “actively targeted” nanomedicines) and/or even simple PEGylation, quickly increases the count of the components to three or more. Among the most prescribed drugs in human history, mRNA vaccines for COVID‐19 are in the latter category. Nanomedicines have been proposed that encapsulate more than one drug and/or drugs plus imaging contrast agents and/or permeation enhancers, further adding to the number of components. The majority of current clinical nanodrugs comprise a complexation of an active agent with a carrier protein or other biological macromolecules.

Even beyond the domain of nanovectored API, much of the innovation in the current pharmaceutical world is empowered by the nanoengineering of multi‐component complexation strategies. Examples include lipid‐derivatized GLP‐1 agonists or insulin, polymer‐conjugated long‐acting psychiatric drugs, the oncology‐omnipresent antibody‐drug conjugates (no less than three‐components, including the linker, which largely determines biodistribution), bi‐(and higher)specific antibodies, as well as siRNA‐based agents that comprise stabilization and targeting moieties. Among the most recent composite therapeutic agents, which comprise an even higher number of components, are multi‐stage vectored drugs (MSV), injectable nanoparticle generators (iNPG), and Hapten or more broadly conjugated vaccines, with or without delivery vectors. MSV and iNPG are starting to mimic some key operational principles of the immune system by functionally linking micro‐ and nano‐scale components in a single therapeutic entity.

A complete rendition of these nanodrug concepts exceeds the scope of this article. However, even casual observation reveals the rationale behind the emergence and exponential growth of multi‐component (composite), typically nano‐engineered therapeutics: the need for optimally combining the specificity of action of the “active principle” with a suitable biodistribution of the agent. The paradox of monoclonal antibodies is a paradigm for this double necessity; while they provide exquisite specificity of action, their distribution to target cancer tissue is at least one order of magnitude less favorable than small molecule chemotherapeutics, which conversely suffer from a less desirable cytotoxic specificity. Thus, the need for multi‐componentry in advanced therapeutic agents—and, one may add—a clear case to finally abandon archaic distinctions between “active principle” and “formulation” or “delivery vectors.” They are both equally needed and form a single, unique agent in contemporary, nanoengineered multicomponent drugs.

### 4.2 Vision for Characterization of New, More Complex Concepts

Most analytical techniques used today are ensemble methods, that is, methods which measure the average or bulk properties. For example, DLS provides the overall size of a sample, but no information on particle concentration within a size population. RP‐HPLC can provide total drug concentration but does not afford information on how that drug is distributed in a polydispersed sample. Techniques such as AF4 combined with in‐line detectors and post‐analysis of collected fractions can help answer these questions but fall short when trying to measure how many nanoparticles are empty versus drug‐loaded. As the nanomedicine field advances, so does the complexity of the nanoformulations, meaning more advanced analytical techniques/methods are needed.

The advanced characterization technologies being developed today are aiming to improve and enhance the understanding of nanomedicines. These techniques strive to improve the resolving power for differentiation and separation of a mixture of size populations, measure the ratio of non‐drug‐loaded versus drug‐loaded nanoparticles, quantitate drug loaded in single nanoparticles/single size populations, assess the distribution of drugs across various size populations, determine the amount of free drug, and finally evaluate the release of drug in plasma or other relevant biological matrices (Clogston 2021). Current technologies such as AF4 are being modified to help address these challenges (Caputo et al. 2021; Grossman et al. 2017; Hu et al. 2020), whereas promising techniques such as simultaneous multi‐laser nanoparticle tracking analysis (Wells et al. 2024), microfluidic technology combined with fluorescence (Pleet et al. 2023; Varga et al. 2020), single particle automated Raman trapping analysis (Penders et al. 2021; Penders et al. 2018), and mass photometry (Foley et al. 2021; Kowal et al. 2024) are being developed, optimized, and tested for these applications. If successful, these emerging techniques will help to provide a more complete physicochemical characterization of the evolving nanomedicine landscape.

The future of immunotoxicity testing of nanoparticles is equally exciting. As the nanomedicine community continues to explore more advanced immunotherapy applications, analysis of non‐endotoxin contaminants becomes more important than ever and is expected to represent a substantial challenge. This expectation is based not only on the complexity of nanomaterials and nanotechnology‐based formulations posing a broad spectrum of interferences with in vitro detection assays—as has been learned using endotoxin and beta‐glucan detection via Limulus Amoebocyte Lysate (LAL)‐based assays—but also the lack of well‐characterized reference innate immune response modulating impurities (IIRMIs), non‐cell‐based but functional (i.e., able to detect and quantify biological activity) assays specific to the given IIRMI, and threshold safety or pyrogenic dose information for each IIRMI. Going forward, even endotoxin detection in nanomaterials will become more complicated as the overall biotechnology community is shifting from traditional LAL to recombinant LAL assays (Bolden et al. 2020; Burgmaier et al. 2024; Di Paolo et al. 2024; Dubczak et al. 2021; Kang et al. 2024; Schromm et al. 2024; Tindall et al. 2021) associated with reproducibility challenges, which are expected to be further complicated due to the breadth of nanomaterials' physicochemical properties. Another recent change affecting the entire drug development field is the FDA Modernization Act 2.0, encouraging a reduction in animal testing (Han 2023). This change increases the importance of in vitro–in vivo correlation studies and is expected to lead to new assays and optimization of traditional assays to screen for nanoparticle‐mediated toxicities. Moving in this direction also opens ample opportunities for artificial intelligence, organ‐on‐a‐chip, artificial tissues, and co‐culture systems. The NCL has already undertaken some such efforts (Cedrone et al. 2024; Chandler et al. 2022; Dobrovolskaia and McNeil 2013b; Potter et al. 2024), and more studies are expected to follow.

For nanomedicine pharmacokinetics, future efforts are expected to include the development of methods to measure nanomedicine drug fractions in tissue to better define pharmacokinetic‐pharmacodynamic relationships (Meng et al. 2025). Also important is the development of new high‐resolution imaging technologies to better characterize shifts in drug distribution and tissue exposure resulting from targeted nanoformulations, such as cryo‐fluorescence tomography (Leach et al. 2024). In the area of active targeting of nanoformulations to sites of disease for drug delivery and diagnosis, there is a need for better disease markers with high selectivity (Crist et al. 2021).

## 5 Conclusion

Over the last two decades, great strides have been made in many areas of nanomedicine, including cancer nanotechnology. Nanomedicine research and particle characterization methodologies continue to evolve; the advances being made in nanoparticle platform design, a deeper understanding of cancer biology, mechanistic insights into various modes of action, vast improvements in instrumentation, aid of artificial intelligence, and more all have the potential to impact the field in dramatic ways and provide exciting new advancements in hopes of diminishing cancer's effects on so much of the population.

## Author Contributions

**Rachael M. Crist:** writing – original draft (lead), writing – review and editing (lead). **Yechezkel Barenholz:** writing – original draft (supporting). **Ahuva Cern:** writing – original draft (supporting). **Kate N. Clark:** writing – original draft (supporting). **Pieter R. Cullis:** writing – original draft (supporting). **Cheryl Dean:** writing – original draft (supporting). **Neil Desai:** writing – original draft (supporting). **Mauro Ferrari:** writing – original draft (supporting). **Matthieu Germain:** writing – original draft (supporting). **Carmen A. Giacomantonio:** writing – original draft (supporting). **Emma Grabarnik:** writing – original draft (supporting). **Piotr Grodzinski:** writing – original draft (supporting). **Atara Hod:** writing – original draft (supporting). **Barry E. Kennedy:** writing – original draft (supporting). **Ruvanthi N. Kularatne:** writing – original draft (supporting). **Glen S. Kwon:** writing – original draft (supporting). **Emmanuel Loeb:** writing – original draft (supporting). **Erin B. Noftall:** writing – original draft (supporting). **Len Pagliaro:** writing – original draft (supporting). **Morteza Rasoulianboroujeni:** writing – original draft (supporting). **Alexander Roth:** writing – original draft (supporting). **Darren Rowles:** writing – original draft (supporting). **Kulbir Singh:** writing – original draft (supporting). **Nicole F. Steinmetz:** writing – original draft (supporting). **Zhanna Yehtina:** writing – original draft (supporting). **Yao Zhang:** writing – original draft (supporting). **Daniel Zilbersheid:** writing – original draft (supporting). **Jeffrey D. Clogston:** writing – original draft (lead). **Stephan T. Stern:** writing – original draft (lead). **Marina A. Dobrovolskaia:** conceptualization (lead), writing – original draft (lead).

## Conflicts of Interest

N.F.S. declares the following competing financial interests: co‐founder and CEO of and has equity in PlantiosX Inc.; co‐founder of and has equity in Mosaic ImmunoEngineering Inc.; co‐founder and manager of Pokometz Scientific LLC, under which she is a paid consultant to Flagship Labs 95 Inc. P.R.C. has a financial interest in Acuitas Therapeutics and NanoVation Therapeutics as well as being Chair of NanoVation Therapeutics. M.F. has a financial interest and affiliation with BrYet US and Arrowhead Pharmaceuticals. M.G. is an employee of Nanobiotix SA. Y.B. and A.C. are coinventors on a patent entitled Liposomal formulations comprising at1 receptor blockers.

## Related WIREs Articles

Improved cancer immunotherapy strategies by nanomedicine

Current landscape of treating different cancers using nanomedicines: Trends and perspectives

## Data Availability

Data sharing is not applicable to this article.
